# Neutralizing Antibodies against 10 SARS-CoV-2 Variants at Two Years Post-COVISHIELD Vaccination with Special Reference to Omicron Subvariants and Booster Administration

**DOI:** 10.3390/vaccines12091039

**Published:** 2024-09-11

**Authors:** Rajashree Patil, Sonali Palkar, Akhileshchandra Mishra, Vidya Arankalle

**Affiliations:** 1 Department of Translational Virology, Interactive Research School for Health Affairs, Bharati Vidyapeeth (Deemed to be) University, Pune 411043, Maharashtra, India; 2 Department of Pediatrics, Bharati Vidyapeeth Medical College, Bharati Vidyapeeth (Deemed to be) University, Pune 411043, Maharashtra, India

**Keywords:** SARS-CoV-2 variants, MSD, omicron, booster, two-year follow-up

## Abstract

To study the durability of neutralizing antibodies (NAbs) against ten SARS-CoV-2 variants among COVISHIELD vaccine recipients from Pune, India, 184 vaccinee samples with (pre-positives) or without (pre-negatives) prior antibody positivity were evaluated. To estimate NAb levels, a validated ten-plex MSD ACE2 neutralization assay was used. NAbs against Alpha, Beta, Delta, and Omicron/subvariants were assessed at 1 month (PD2-1) and 6 months (PD2-6) post-vaccination, post-booster dose, and 2 years (2Y) post-vaccination. In pre-negatives, the seropositivity declined from PD2-1 to PD2-6 for all variants (Omicron variants: 14–54% to 0%; non-Omicron variants: 66–100% to 8–44%). In pre-positives, the decline in seropositivity from PD2-1 to PD2-6 was seen only for Omicron variants (14–39%). At PD2-6, a significant reduction in NAb levels was observed in all vaccinees against all the variants. Irrespective of prior exposure, the diminished anti-variant antibody levels at PD2-6 increased significantly following the administration of the booster. In conclusion, the COVISHIELD vaccine booster dose did provide cross-neutralizing antibodies against broad-range SARS-CoV-2 variants with improved durability up to [16 (15–18)] months post-booster dose and two years post-vaccination.

## 1. Introduction

Severe acute respiratory syndrome coronavirus-2 (SARS-CoV-2), with different variants, has been circulating all over the world for around four years. Vaccination of populations played a significant role in controlling the COVID-19 pandemic [1]. In India, ~82% of the population is vaccinated with the COVISHIELD vaccine, with two dose regimens. However, the decline in vaccine-elicited antibody levels over time and the emergence of SARS-CoV-2 variants arose as a major worry about the durability of vaccine-induced immunity [2,3,4]. Hence, a booster dose was recommended to maintain neutralizing antibody levels. The government of India introduced a booster dose referred to as the “precautionary dose” in January 2022. At first, this option was only available to frontline workers, individuals with underlying health conditions, and individuals aged 60 years and above. However, starting in April 2022, the eligibility for the booster shot was expanded to include all adults across the nation.

The SARS-CoV-2 virus is evolving continuously and generating newer Variants of Concern (VOCs). The newest VOCs are the Omicron variant/subvariants. In mid-November 2021, Omicron variant BA.1 was first identified in South Africa, after which other Omicron subvariants rapidly circulated [5]. The Omicron variant possesses ~30 mutations in the spike protein region that affect transmissibility and immune escaping capabilities [6,7,8]. The increasing number of breakthrough infections caused by Omicron variants raises a concern about COVID-19 vaccine efficacy. Considering the waning of vaccine-induced antibodies and Omicron variants’ immune escape capability, there is a need for continuous monitoring of the vaccine-induced immune response.

Hence, in the current study, we studied COVISHIELD vaccine-induced neutralizing antibodies at different time points up to 25–29 months, referred to as 2 years, and the effect of a booster dose. A validated multiplex ACE2 neutralization assay from Mesoscale discovery was performed to assess the COVISHIELD vaccine-elicited neutralizing antibodies against 10 different SARS-CoV-2 variants including Alpha, Beta, Delta, and Omicron variants. The two-dose regimen was found to be insufficient in sustaining effective antibody levels. The COVISHIELD vaccine booster dose provided cross-neutralizing antibodies against a broad-range of SARS-CoV-2 variants including Omicron variants with improved durability up to [16 (15–18)] months post-booster dose and two years post-vaccination.

## 2. Methodology

### 2.1. Vaccine

COVISHIELD vaccine: The University of Oxford (Oxford, UK) developed ChAdOx1 nCoV-19, the chimpanzee adenoviral vectored vaccine. AstraZeneca, UK, manufactured this vaccine as AZD1222 (the coded research name) and Vaxzevria (the trade name). Later, the Serum Institute of India Pvt Ltd. (SIIPL) manufactured this vaccine in India with “COVISHIELD” as the trade name. One vaccine dose contains 5 × 10^5^ viral particles. COVISHIELD is fully equivalent to the Vaxzevria vaccine.

### 2.2. Study Population and Samples

**Figure 1:**
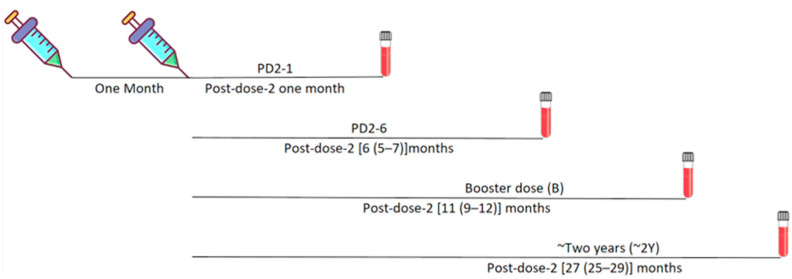
Schematic presentation of COVISHIELD vaccination with the recommended two-dose schedule and the timeline for blood sample collection. Timeline given as [median (minimum–maximum) months].

Serum/plasma samples of healthcare personnel, who completed a full two-dose COVISHIELD vaccine regimen and provided written informed consent, were collected at different time points until two years post-vaccination (Figure 1).

At each time-point, the vaccinee samples were classified into two groups, pre-negative and pre-positive, based on the status of IgG-anti-SARS-CoV-2 antibodies just before vaccination. Follow-up samples were collected at 25–29 months post-two vaccine doses. Henceforth, this time point will be referred to as two years (2Y). The demographic information of the vaccinees is presented in Table 1.

### 2.3. Ethical Approval

This study includes a subset of vaccinee samples from a previous study [3]. The study involving sample collection at two years was approved by the institutional “Human Ethics Committee” (No:BVDUMC/IEC/81). Serum/plasma samples collected earlier from vaccinees and stored in aliquots at −70 °C were used for testing. Due to multiple tests and the limited number of serum specimens, samples for every subject at all pre-determined time points were unavailable.

### 2.4. MSD Assay

An MSD ACE2 competition/neutralization assay was performed using MSD V-PLEX neutralization kits (SARS-CoV-2 panel-25 (ACE2), K15586U-2) as per the instructions of the manufacturer and the detailed methodology mentioned in our previous study [2]. Briefly, precoated multiplex assay plates containing 10 spike antigens of distinct SARS-CoV-2 variants in a single well of a 96-well plate were provided in the MSD V-PLEX neutralization kits (SARS-CoV-2 panel-25 (ACE2), K15586U-2). Initially, the assay plates underwent a blocking process using 150 µL/well of MSD Blocker A solution at ambient temperature with agitation (~700–800 RPM) for a duration of 30 min. Serum specimens were diluted to a 1:10 ratio in MSD-Diluent 100, while the calibrator was diluted according to the provided instructions by the manufacturer. The blocked plates were subjected to three washes with 150 µL of MSD wash buffer. Subsequently, samples (25 µL) and calibrators (25 µL) were dispensed in duplicate onto the plate and incubated for 1 h at room temperature with agitation (~700–800 RPM). Following the incubation period, 1× SULFOTAG human ACE2 protein solution (detection solution) (25 µL) was added to the same wells containing serum samples, and the plates were further incubated with agitation for an additional hour. The SULFO-TAG human ACE2 protein competes with the precoated variants antigen for binding with neutralizing antibodies present in the serum samples. The plates underwent three washes with MSD wash buffer (150 µL per well). Immediately post-washing, MSD GOLD Read Buffer B (150 µL) containing the ECL substrate was applied to the plate. The plates were analyzed using the MESO QuickPlex SQ120 plate imager (Meso Scale Diagnostics, LLC, Rockville, MD, USA).

### 2.5. Statistical Analysis

All statistical analyses were performed in GraphPad Prism 9.5.1. R 4.1.3 was used to calculate statistical significance in % seropositivity. ACE2 competition values were compared using the Kruskal–Wallis test, followed by the post hoc Dunn’s test, as indicated in the respective figure legends. For comparison between PD2-1 and PD2-6, a Wilcoxon matched-pairs signed rank test was performed, and for pre-positive and pre-negative comparison, a two-tailed Mann–Whitney U test was performed.

## 3. Results

Our earlier study used an MSD panel 15 assay to determine neutralizing antibody (Nab) responses against 10 SARS-CoV-2 variants [3]. Subsequently, to understand similar responses against Omicron variants causing a global wave of the disease, we used an MSD-panel 25 assay including B.1.1.529 and subvariants BA.3, BA.2, BA.1+R346K, amd BA.1+L452R, as well as other historically important variants such as Wuhan, Alpha (B.1.1.7), Beta (B.1.351), Delta (B.1.617.2), and B.1.640.2. Fold inhibition obtained with the MSD panel 15 assay could not be directly compared with the MSD panel 25, as both the assays have different antigens/variants. Three variants were common in both assays, but spike mutations differed (difference in variant mutation is shown in this manner, MSD panel 15 variant mutation: B.1.1.7+E484K: *B.1.1.7*; B.1.135.1: *B.1.351*; and B1.617.2+Del Y144: *B.1.617.2*). Still, comparable seropositivity was found in both assays (n = 119) (Supplementary Figure S1). A sample is considered seropositive when the fold inhibition value is >1.5-fold for a particular variant.

To understand the dynamics of variant-specific neutralizing antibodies, samples collected at different time points until two years post-vaccination were tested among pre-negative and pre-positive vaccinees.

### 3.1. Neutralizing Antibody Response against Different Variants until Six Months Post-Vaccination

#### 3.1.1. Seropositivity

**Figure 2:**
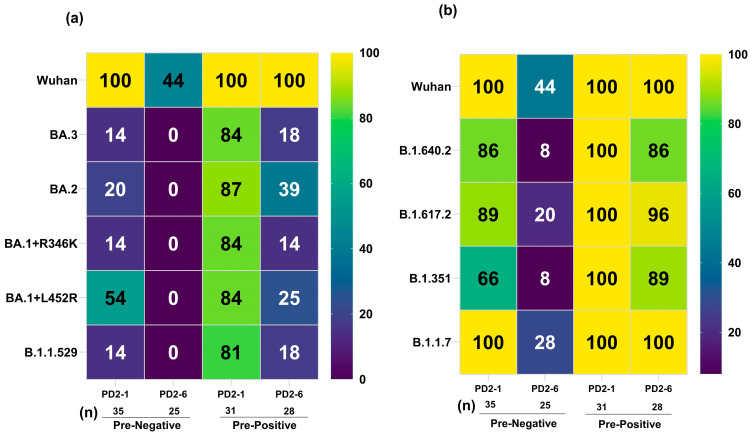
Serum/plasma samples from COVISHIELD vaccinees either negative (pre-negative) or positive (pre-positive) for IgG-anti-SARS-CoV-2 antibodies were tested for anti-variant antibodies in the MSD-25 assay at one month (PD2-1) and six months (PD2-6) post-dose 2. (**a**) Percent seropositivity of Omicron variants and (**b**) non-Omicron variants are shown here. The number of serum/plasma samples in each category is mentioned below each column in the table.

We first calculated percent seropositivity at both time points for Omicron (Figure 2a) and non-Omicron (Figure 2b) variants to study the breadth of variant-specific antibody responses. At one-month post-vaccination (PD2-1) when the peak antibody titers were observed [2,3] the lowest responder rate in the pre-negative vaccinees (n = 35) was noted against BA.3, BA.2, BA.1+R346K, and B.1.1.529 variants (14–20%). In contrast, 54% of the vaccinees were seropositive for the BA.1+L452R variant, suggesting that differential mutations are responsible for the varied responder rates within the Omicron subvariants. Among the non-Omicron variants, B.1.351 with 66% seropositivity was the lowest (*p* < 0.0001). Among the pre-positive vaccinees (n = 31), seropositivity was superior (81–87%) against the Omicron subvariants. Of these, significantly lower (81%, *p* = 0.032) seropositivity was seen against the Omicron variant B.1.1.529 compared to the ancestral Wuhan virus. Of most concern, in the pre-negative vaccinee group, no seropositivity was seen against the Omicron subvariants at 6 months post-vaccination (PD2-6), and a distinct reduction in anti-Wuhan, -Alpha, and -Delta seropositivity (20–44%) was evident; 8% exhibited antibodies against B.1.351 and B.1.640.2 variants. (*p* = 0.01–<0.0001). Clearly, six months post-second dose, the pre-negative vaccinees were negative for antibodies against all the Omicron variants and the majority against the other variants and the Wuhan virus (44% positivity). In the pre-positive vaccinees as well (n = 28), the lowest responder rate (14–39%, *p* = 0.01–<0.0001) was observed for the Omicron variants. However, the degree of reduction was lesser among the pre-positives (*p* = 0.0083–<0.0001). Thus, even most of the naturally infected and immunized individuals did not possess antibodies against the Omicron subvariants.

#### 3.1.2. Anti-Variant Neutralizing Antibody Levels

**Figure 3:**
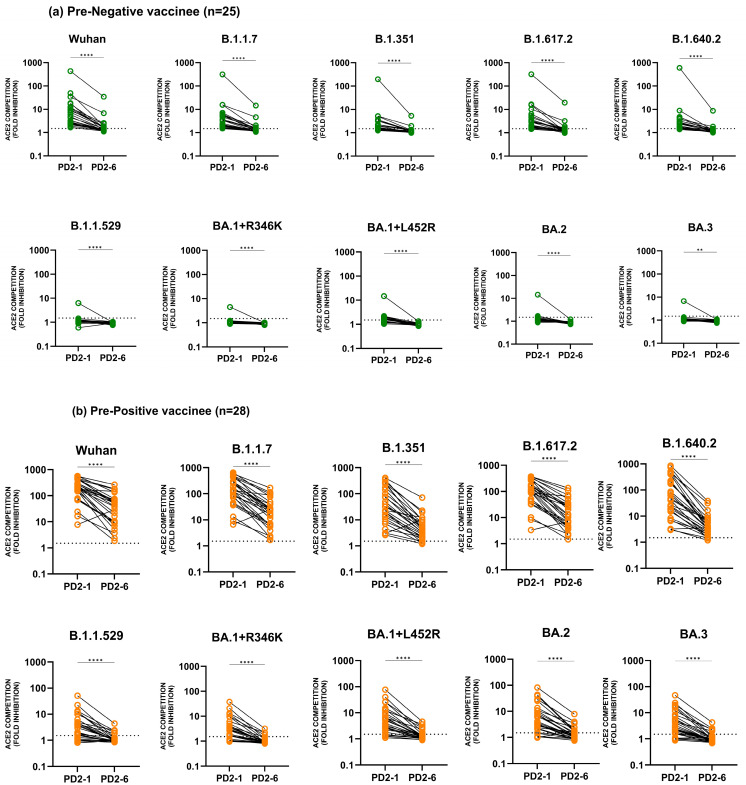
Serum/plasma samples from COVISHIELD vaccinees either (**a**) negative (pre-negative, n = 25) or (**b**) positive (pre-positive, n = 28) for IgG-anti-SARS-CoV-2 antibodies before immunization were tested for anti-variant antibodies in the MSD-25 assay at 1 (PD2-1) and 6 months (PD2-6) post-2nd dose. Wilcoxon matched-pairs signed rank test was used for comparing ACE2 competition (fold inhibition) values at PD2-1 and PD2-6. ** *p* = 0.001 and **** *p* ≤ 0.0001. The dotted line denotes the assay cut-off value (1.5-fold).

Figure 3 depicts the dynamics of anti-Wuhan and -variant antibodies at 1 month and 6 months post-vaccination. The data indicate unequivocally that six months after complete vaccination, there was a significant reduction (*p* = 0.001–<0.0001) in anti-Wuhan and anti-variant antibody levels in the pre-negative (Figure 3a) and pre-positive vaccinees (Figure 3b).

### 3.2. Administration of a Booster Dose and Follow-Up until Two Years Post-Vaccination

In December 2021, the government of India announced the administration of a booster dose termed the “precautionary dose”, healthcare personnel being the priority. This was voluntary. The pre-negatives with drastically reduced titers at 6 months opted for booster. However, as pre-positives exhibited high titers following vaccination (PRNT test reports given to the individual healthcare worker), the proportion of pre-positives opting for the booster dose was low. As the booster dose was received at different times as per the convenience of the vaccinee, the follow-up at this point was not satisfactory (16/35, 45.7% and 7/31, 22.5% respectively).

#### 3.2.1. Seropositivity Post-Booster and at 2 Years Post-Vaccination

**Figure 4:**
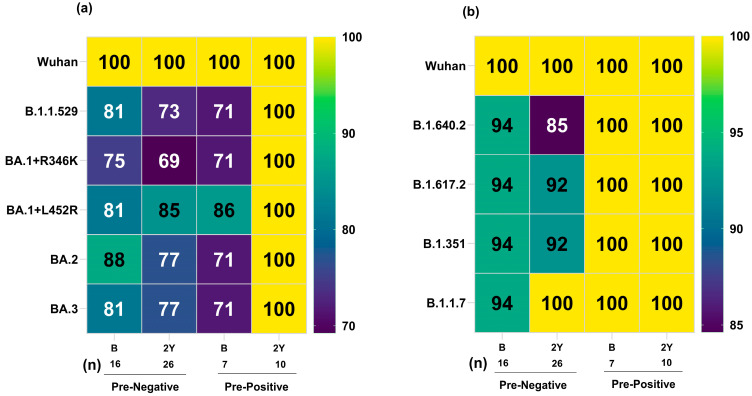
Serum/plasma samples from COVISHIELD vaccinees either negative (pre-negative) or positive (pre-positive) for IgG-anti-SARS-CoV-2 antibodies were tested for anti-variant antibodies in the MSD-25 assay at post-booster dose (B) and 2 years (2Y) post-2nd dose. (**a**) Percent seropositivity of Omicron variants and (**b**) non-Omicron variants are shown here. The number of serum/plasma samples in each category is mentioned below each column in the table.

The administration of the booster dose led to high seropositivity against Omicron (75–88% in pre-negatives and 71–86% in the pre-positives) as well as all the other variants (94% in pre-negatives and 100% in pre-positives) (Figure 4a,b). These results emphasize the need for either three doses or an early booster to protect against the emerging variants. At two years post-vaccination, we could collect samples from 42 vaccinees. Of these, 26/27 of the pre-negatives and 10/15 of the pre-positives were booster dose recipients. Due to small numbers, non-boosters were removed from further analysis. Thus, we could follow 27/35 (77.1%) pre-negatives and 15/31 pre-positive (48.3%) vaccinees for two years. Seropositivity of 69–77% (pre-negatives) and 100% (pre-positives) was maintained against BA.3, BA.2, BA.1+R346K, and B.1.1.529 variants. For all the remaining variants, 85–100% and 100% seropositivity continued in the pre-negative and pre-positive groups, respectively. The administration of a booster dose was crucial in regaining diminished seropositivity at PD2-6, which was reasonably high when tested at two years post-vaccination. The utility of a booster dose in general, and that among pre-negatives in particular, was evident for maintaining antibody response until 2 years post-vaccination.

#### 3.2.2. Neutralizing Antibody Levels Post-Booster and at Two Years Post-Vaccination

**Figure 5:**
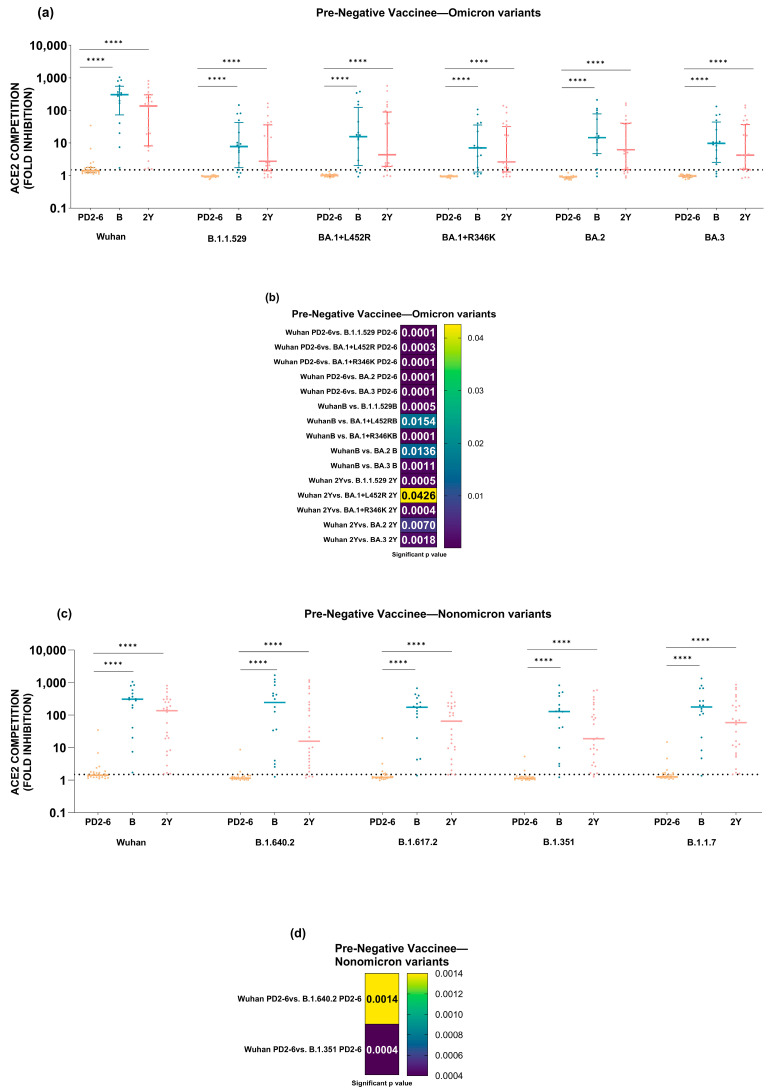
Serum/plasma samples from pre-negative COVISHIELD vaccine recipients were tested for anti-variant antibodies in the MSD-panel 25 assay at PD2-6 (n = 25), post-booster (n = 16), and 2 years (n = 26). The cutoff value for the assay was >1.5. (**a**) (Omicron variants) and (**c**) (non-Omicron variants) depict ACE2 competition (fold inhibition) values compared at different time points for each variant. (**b**) (Omicron variants) and (**d**) (non-Omicron variants) describe significant *p* values when fold inhibition values were compared between variants to the Wuhan strain at respective times. Statistical comparison was performed by using the Kruskal–Wallis test (post hoc test—Dunn’s multiple comparison test). The variation in the graph measures the interquartile range (25–75%). **** represents *p* value < 0.0001.

**Figure 6:**
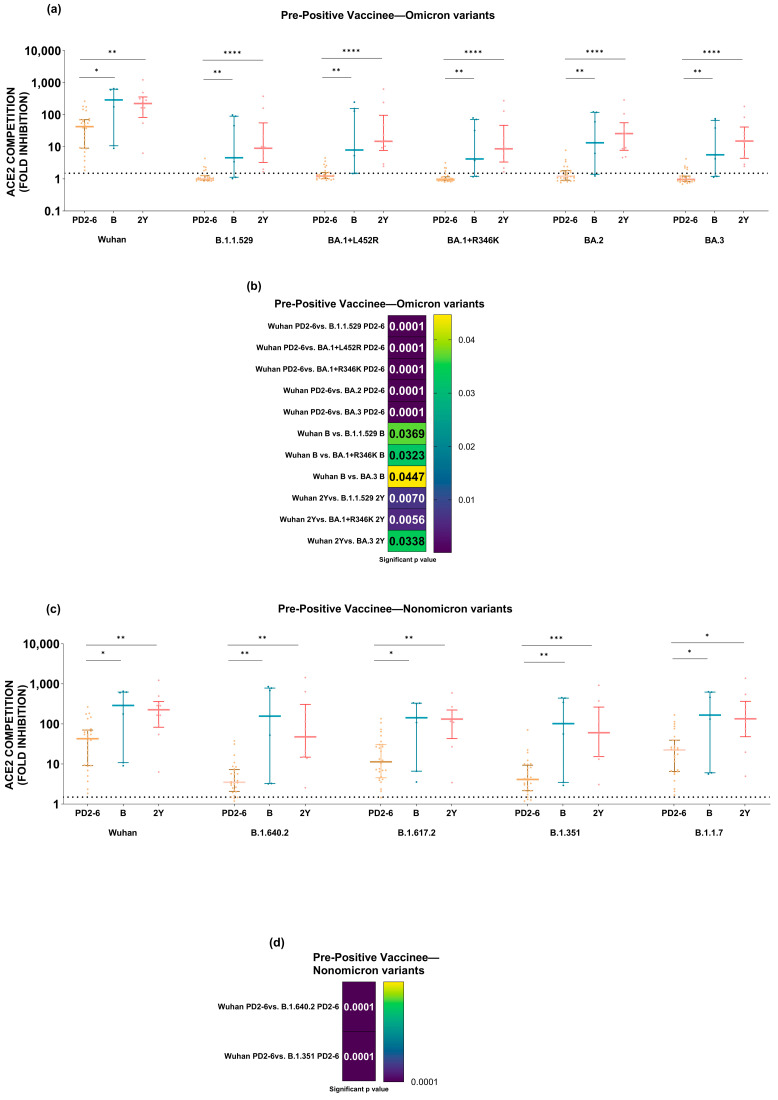
Serum/plasma samples from pre-positive COVISHIELD vaccine recipients were tested for anti-variant antibodies in the MSD-panel 25 assay at PD2-6 (n = 28), post-booster (n = 7) and at 2-years (n = 10). The cut-off value for the assay was >1.5. (**a**) (Omicron variants) and (**c**) (non-Omicron variants) depict ACE2 competition (fold inhibition) values compared at different time points for each variant. (**b**) (Omicron variants) and (**d**) (non-Omicron variants) describe significant *p* values when fold inhibition values were compared between variants to the Wuhan strain at respective times. Statistical comparison was performed by using the Kruskal–Wallis test (post hoc test—Dunn’s multiple comparison test). The variation in the graph measures the interquartile range (25–75%) and stars express *p* values as follows: **** *p* < 0.0001, *** *p* < 0.001, ** *p* < 0.01, and * *p* < 0.05.

To assess this, the neutralization capacity of COVISHIELD vaccine-induced antibodies as evidenced by ACE2 competition at PD2-6 was compared with two subsequent time points among pre-negatives (Figure 5) and pre-positives (Figure 6). The analysis confirmed that the administration of a booster dose to the pre-negatives led to a significant rise in neutralizing antibody levels against all the variants that was drastically reduced at PD2-6. Furthermore, the retention of the booster effect continued for at least two years post-vaccination, re-emphasizing the inadequacy of the current schedule and the necessity of a booster dose (Figure 5a,c). Comparisons of the kinetics of anti-variant antibodies revealed that ACE2 competition of Omicron variants was significantly reduced compared to the Wuhan strain at all the time points examined (*p* values = 0.04–0.0001) (Figure 5b). For non-Omicron variants, when compared to the Wuhan strain, a significant reduction at PD2-6 was seen only for the B.1.640 and B.1.351 variants (*p* values = 0.001–0.0004) (Figure 5d).

Like the pre-negatives, post-booster antibody levels in pre-positives increased for all the variants compared to the respective levels at PD2-6 and were maintained for at least 2 years (Figure 6a,c). Except for the BA.2 variant, the levels of anti-Omicron antibodies were significantly lower compared to the Wuhan strain at both subsequent times (*p* values = 0.04–0.0001) (Figure 6b). Among the non-Omicron variants, anti-variant antibody levels were reduced for all the variants except B.1.1.7 and B.1.617.2 (Figure 6d). Although natural infection before vaccination was beneficial, the generated antibodies were insufficient when Omicron subvariants were considered. Irrespective of the status before vaccination, the diminished anti-variant antibody levels at PD2-6 increased significantly following the administration of the booster (Figure 5 and Figure 6).

### 3.3. Comparison of Anti-Variant Antibody Levels among Pre-Negatives and Pre-Positives

**Figure 7:**
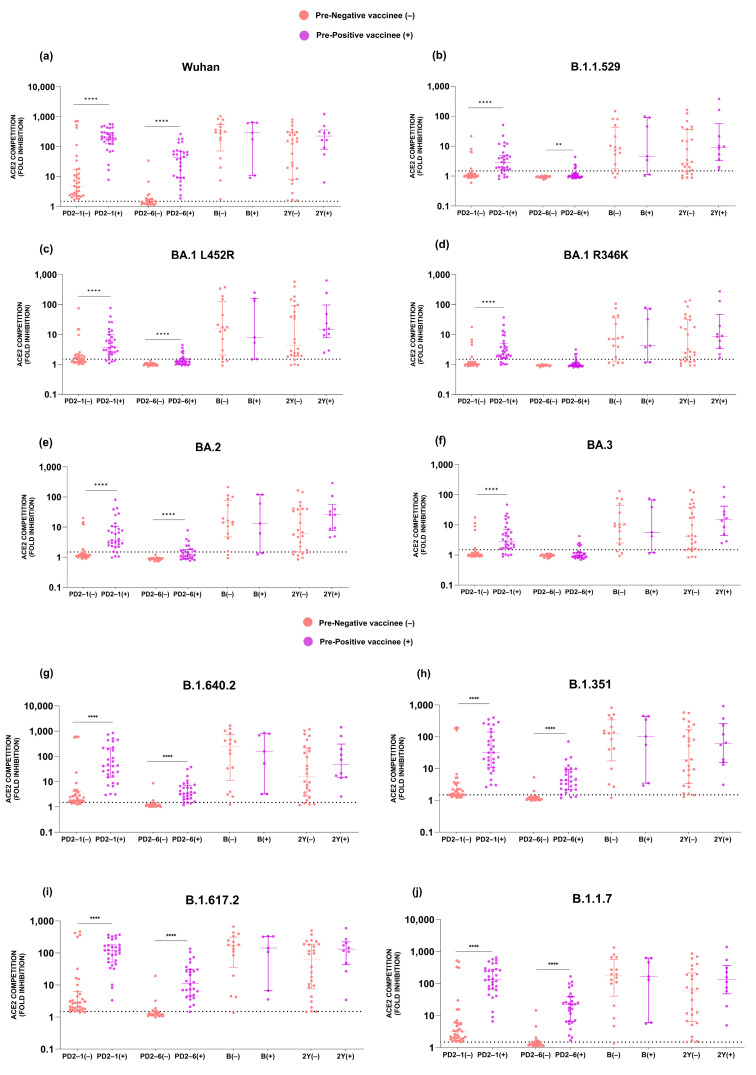
ACE2 competition (fold inhibition) values were compared among pre-negative (−) and pre-positive (+) vaccinees at all the time points. (**a**–**j**) Variant-specific comparisons at individual time points among pre-negatives and pre-positives were performed using the Mann–Whitney U test. **** *p* < 0.0001, ** *p* < 0.01. The value above each bar represents the median ACE2 competition value; variations measure the interquartile range (25–75%).

At PD2-1, anti-Wuhan and anti-Omicron antibodies in the pre-negative vaccinees were significantly lower than in the pre-positive group (Figure 7a–j). At PD2-6, neutralizing antibodies against Wuhan, BA.2, BA.1+L452R, and B.1.1.529 variants were significantly reduced in the pre-negative vaccinees (Figure 7a–c,e). Further, in both the groups, anti-BA.3 and anti-BA.1+R346K antibodies were below the detection limit (1.5 fold) (Figure 7d,f). Like our previous observations at PD2-1 and PD2-6, antibody levels against all the non-Omicron variants in the pre-negative category were significantly lower than in the pre-positives [3]. Of major significance, irrespective of prior infection, the booster dose recipients circulated comparable anti-variant antibodies when tested at two years post-vaccination (Figure 7g–j).

## 4. Discussion

We have provided a comprehensive analysis of the immune response generated against multiple non-Omicron and Omicron subvariants among COVISHIELD vaccine recipients and follow-up until 2 years post-vaccination. This is an extension of our previous study wherein 6-month follow-up samples were tested against earlier non-Omicron variants [3]. We showed that the MSD assay was comparable with the plaque reduction neutralization test employing live wild-type virus (D614G). A different panel for Omicron testing was used for the present study and hence we first compared the results for the variants common to both panels. Comparability of both assays implies that the antibodies detected do have neutralization potential. As the predominant vaccine used in India, our study is restricted to COVISHIELD. A significant population proportion was vaccinated with vaccines employing different platforms by the time Omicron and its subvariants emerged globally. Several studies have reported the diminished response of vaccine-induced antibodies against non-Omicron and Omicron variants [4,5,6,7]. Irrespective of the type of the vaccine, the maximum impact was on Omicron/subvariants. ChAdOx1-S and BNT162b2 boosters in older adults were shown to protect against symptomatic disease and hospitalization with Omicron/Delta variants in England [9]. The study suggested that ChAdOx1-S booster receivers do not require re-vaccination ahead of others.

Our data confirm and emphasize that the current schedule of two doses is not useful in protecting from SARS-CoV-2 variants and a booster or a third dose is essential. When we compare our observations with the same vaccine (ChAdOx1-S/Vaxzevria/COVISHIELD/AstraZeneca) used elsewhere, the overall results are comparable or at variance. When a pseudovirus-based neutralization assay was carried out in Germany, the AZD1222 vaccine had a 56% responder rate against BA.1 [10]. In our MSD-based study, the responder rates with the COVISHIELD vaccine were 54% (BA.1+L452R) and 14% (BA.1+R346K). The drastic reduction in the responder rate after acquiring a single mutation within a subvariant is noteworthy and demands the requirement of second-generation vaccines with broader neutralization capacity. In Thailand, among the vaccinees receiving AZD1222/AZD1222, the proportion of detectable NAb-Omicron (27%) was considerably lesser than those of NAb-WT (89.2%) when 120 participants were considered [11]. Though our sample size was 35, the trend of responder rates is similar; 100% (WT) and 14% (Omicron). Omicron subvariants were not considered in the Thailand study.

Though with a small sample size, the efficacy of a COVISHIELD booster was obvious. The diminished antibody response at 6 months was quickly restored for both Omicron and non-Omicron variants among pre-negatives, with 100% of the pre-positives possessing NAbs against all the variants. This was indeed very encouraging, as a homologous booster was administered. It is pertinent to note here that heterologous booster administration has been shown to provide superior antibody responses [12,13]. Interestingly, in both pre-negatives and -positives, the titers against the non-Omicron variants were like the ancestral strain, but lower for Omicron and its subvariants. Metric and ultra-metric methods showed that the Omicron variant forms a separate monophyletic clade and is not a descendant of the Delta variant or the ancestral strain [14,15]. The immune escape of Omicron variants is attributed to 15 mutations in the receptor-binding domain (RBD) and ~30 mutations in the spike protein [16]. Our observations point out that within the BA.1 subvariant, the alteration of a single amino acid could reduce the responder rate from 54% (BA.1+L452R) to 14% (BA.1+R346K), which was like the Omicron variant and BA.2/BA.3 subvariants. A key mutation, L455S in spike protein enhanced immune evasion in JN.1 than BA.2.86 [17]. Such observations underscore a definite need for continuous monitoring of SARS-CoV-2 despite the current drop in clinical cases.

The unique finding of our study comes from the follow-up of 42 vaccinees until 2 years post-vaccination. Of these, 27 were from the pre-negative group and 26 received the booster dose. Among the 15 pre-positives, 10 received the booster dose. NAb levels against all the variants were comparable among the booster recipients, irrespective of exposure to the virus before vaccination. This is indeed reassuring and there is a need to compare the impact of different timings of booster dose on antibody levels. In our case, the booster dose was administered as soon as the government of India announced the policy (6–6.5 months post six months of vaccination).

For the persistence of antibodies and protection, neutralizing antibodies and cellular responses are important. Being cross-reactive, these responses play a crucial role when the host encounters a variant. Previously, we demonstrated that 77.8% of COVISHIELD recipients elicited T-cell responses [2]. Of note, 4/6 non-responders elicited spike-specific T cell response. By six months, though neutralizing antibodies reduced drastically, T cell response remained unaltered and similar in both pre-negatives and pre-positives. This could be one of the contributory factors for the observed boosting effect and improved NAb reactivity/levels against Omicron and non-Omicron variants. Notably, similar observations of unchanged T-cell and reduced antibody titers have been reported for the BNT162b2 vaccine [18,19].

Our study has certain limitations. We did not collect pre-booster samples and hence the precise effect of the booster could not be measured; the number of post-booster samples was low.

In conclusion, neutralizing antibody levels against the ancestral strain and Omicron/non-Omicron variants decline rapidly by six months. Even at the optimum NAb response at PD2-1, the anti-Omicron response among pre-negatives was negligible. However, COVISHIELD booster administration enhanced NAb levels against Omicron and non-Omicron variants, and prior exposure to the virus before vaccination did not impact this rise until 2 years later. Post-pandemic variant surveillance must be continued to identify unique variants and the development of second-generation vaccines is the need of the hour as part of the readiness to deal with any eventuality.

## Figures and Tables

**Table 1:** Demographic information of COVISHIELD vaccinee (n = 184).

	One Month Post 2nd Dose of Vaccine (PD2-1)		Six Months Post 2nd Dose of Vaccine (PD2-6)		Booster Dose Post-Vaccination (B)		2 Years Post-Vaccination—PRE-NEGATIVE		2 Years Post-Vaccination—PRE-POSITIVE	
	PRE-NEGATIVE	PRE-POSITIVE	PRE-NEGATIVE	PRE-POSITIVE	PRE-NEGATIVE	PRE-POSITIVE	Booster Dose Taken (2Y-B+)	Booster Dose Not Taken (2Y-B-)	Booster Dose Taken (2Y-B+)	Booster Dose Not Taken (2Y-B-)
N	35	31	25	28	16	7	26	1	10	5
Female (%)	22 (63)	15 (48)	17 (68)	14 (50)	11 (68.75)	4 (57)	15 (58)	1 (100)	5 (50)	1 (20)
Male (%)	13 (37)	16 (52)	8 (32)	14 (50)	5 (31.25)	3 (43)	11 (42)	0 (0)	5 (50)	4 (80)
Age in years										
Mean	42	35	43	36	43	38	43	26	39	32
Median	44	33	45	35	41	37	40	26	39	29
Range	50	31	50	30	43	21	54	0	28	16
Sample type	Plasma	Plasma	Serum	Serum	Plasma	Plasma	Plasma	Plasma	Plasma	Plasma

## Data Availability

Data will be provided on request.

## Supplementary Materials

The following supporting information can be downloaded at: https://www.mdpi.com/article/10.3390/vaccines12091039/s1, Figure S1: Comparison between seropositivity of MSD panel 15 and MSD panel 25.

## References

[1] Fontanet A., Autran B., Lina B., Kieny M.P., Karim S.S.A., Sridhar D. (2021). SARS-CoV-2 Variants and Ending the COVID-19 Pandemic. Lancet.

[2] Arankalle V., Kulkarni-Munje A., Kulkarni R., Palkar S., Patil R., Oswal J., Lalwani S., Mishra A.C. (2022). Immunogenicity of Two COVID-19 Vaccines Used in India: An Observational Cohort Study in Health Care Workers from a Tertiary Care Hospital. Front. Immunol..

[3] Patil R., Palkar S., Mishra A., Patil R., Arankalle V. (2023). Variable Neutralizing Antibody Responses to 10 SARS-CoV-2 Variants in Natural Infection with Wild- Type (B.1) Virus, Kappa (B.1.617.1), and Delta (B.1.617.2) Variants and COVISHIELD Vaccine Immunization in India: Utility of the MSD Platform. Front. Immunol..

[4] Haritay S., Patil R., Maldar A., Kumar A., Reddy V., Oswal D., Tahashildar M.A., Kolakar A., Kabbur S., Prasad J.B. (2023). Waning of Antibody Response Among Vaccinees Who Received Two Doses of Covishield Vaccine. J. Glob. Infect. Dis..

[5] Tegally H., Moir M., Everatt J., Giovanetti M., Scheepers C., Wilkinson E., Subramoney K., Makatini Z., Moyo S., Amoako D.G. (2022). Emergence of SARS-CoV-2 Omicron Lineages BA.4 and BA.5 in South Africa. Nat. Med..

[6] Greaney A.J., Starr T.N., Gilchuk P., Zost S.J., Binshtein E., Loes A.N., Hilton S.K., Huddleston J., Eguia R., Crawford K.H.D. (2021). Complete Mapping of Mutations to the SARS-CoV-2 Spike Receptor-Binding Domain That Escape Antibody Recognition. Cell Host Microbe.

[7] Harvey W.T., Carabelli A.M., Jackson B., Gupta R.K., Thomson E.C., Harrison E.M., Ludden C., Reeve R., Rambaut A., Peacock S.J. (2021). SARS-CoV-2 Variants, Spike Mutations and Immune Escape. Nat. Rev. Microbiol..

[8] Karim S.S.A., Karim Q.A. (2021). Omicron SARS-CoV-2 Variant: A New Chapter in the COVID-19 Pandemic. Lancet.

[9] Cordelia F., Kirsebom M., Andrews N., Sachdeva R., Stowe J., Ramsay M., Lopez Bernal J. (2022). Effectiveness of ChAdOx1-S COVID-19 Booster Vaccination against the Omicron and Delta Variants in England. Nat. Commun..

[10] Jacobsen H., Strengert M., Maaß H., Ynga Durand M.A., Katzmarzyk M., Kessel B., Harries M., Rand U., Abassi L., Kim Y. (2022). Diminished Neutralization Responses towards SARS-CoV-2 Omicron VoC after MRNA or Vector-Based COVID-19 Vaccinations. Sci. Rep..

[11] Sudjaritruk T., Mueangmo O., Saheng J., Winichakoon P., Salee P., Wongjak W., Chaito T., Praparattanapan J., Nuket K., Solai N. (2023). Comparison of Immunogenicity and Reactogenicity of Five Primary Series of COVID-19 Vaccine Regimens against Circulating SARS-CoV-2 Variants of Concern among Healthy Thai Populations. Vaccines.

[12] Costa Clemens S.A., Weckx L., Clemens R., Almeida Mendes A.V., Ramos Souza A., Silveira M.B.V., da Guarda S.N.F., de Nobrega M.M., de Moraes Pinto M.I., Gonzalez I.G.S. (2022). Heterologous versus Homologous COVID-19 Booster Vaccination in Previous Recipients of Two Doses of CoronaVac COVID-19 Vaccine in Brazil (RHH-001): A Phase 4, Non-Inferiority, Single Blind, Randomised Study. Lancet.

[13] Rose W., Raju R., Babji S., George A., Madhavan R., Leander Xavier J.V., David Chelladurai J.S., Nikitha O.S., Deborah A.A., Vijayakumar S. (2023). Immunogenicity and Safety of Homologous and Heterologous Booster Vaccination of ChAdOx1 NCoV-19 (COVISHIELDTM) and BBV152 (COVAXIN^®^): A Non-Inferiority Phase 4, Participant and Observer-Blinded, Randomised Study. Lancet Reg. Health.

[14] Ren S.-Y., Wang W.-B., Gao R.-D., Zhou A.-M. (2022). Omicron Variant (B.1.1.529) of SARS-CoV-2: Mutation, Infectivity, Transmission, and Vaccine Resistance. World J. Clin. Cases.

[15] Kupferschmidt K. (2021). Where Did “weird” Omicron Come From?. Science.

[16] Wang W.B., Ma Y.B., Lei Z.H., Zhang X.F., Li J., Li S.S., Dong Z.Y., Liang Y., Li Q.M., Su J.G. (2023). Identification of Key Mutations Responsible for the Enhancement of Receptor-Binding Affinity and Immune Escape of SARS-CoV-2 Omicron Variant. J. Mol. Graph. Model..

[17] Yang S., Yu Y., Xu Y., Jian F., Song W., Yisimayi A., Wang P., Wang J., Liu J., Yu L. (2024). Fast Evolution of SARS-CoV-2 BA.2.86 to JN.1 under Heavy Immune Pressure. Lancet Infect. Dis..

[18] Kato H., Miyakawa K., Ohtake N., Yamaoka Y., Yajima S., Yamazaki E., Shimada T., Goto A., Nakajima H., Ryo A. (2022). Vaccine-induced humoral response against SARS-CoV-2 dramatically declined but cellular immunity possibly remained at 6 months post BNT162b2 vaccination. Vaccine.

[19] Bonnet B., Chabrolles H., Archimbaud C., Brebion A., Cosme J., Dutheil F., Lambert C., Junda M., Mirand A., Ollier A. (2022). Decline of Humoral and Cellular Immune Responses Against SARS-CoV-2 6 Months After Full BNT162b2 Vaccination in Hospital Healthcare Workers. Front. Immunol..

