# Supplementary figures and images for: Neutralizing Antibodies against 10 SARS-CoV-2 Variants at Two Years Post-COVISHIELD Vaccination with Special Reference to Omicron Subvariants and Booster Administration
Source: Vaccines (Basel). 2024 Sep 11;12(9):1039. doi: 10.3390/vaccines12091039 (PMC11435521; doi:10.3390/vaccines12091039)

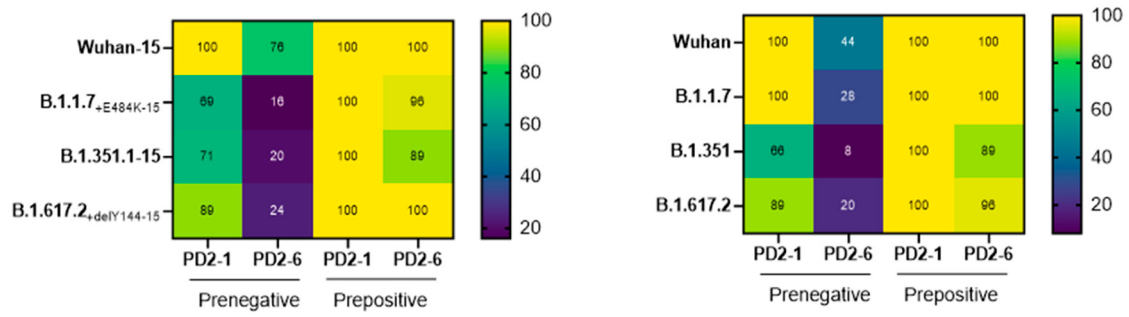

**Figure S1.** Comparison between seropositivity of MSD panel 15 and MSD panel 25.

Supplement: Supplementary file 1 [file vaccines-12-01039-s001.zip › vaccines-3036642-supplementary.pdf]
